# Analysis of Whole-Body Vibration Using Electric Powered Wheelchairs on Surface Transitions

**DOI:** 10.3390/vibration5010006

**Published:** 2022-01-30

**Authors:** Jorge L. Candiotti, Ahlad Neti, Sivashankar Sivakanthan, Rory A. Cooper

**Affiliations:** 1 US Department of Veterans Affairs, Pittsburgh, PA 15206, USA;; 2 Bioengineering Department, School of Engineering, University of Pittsburgh, Pittsburgh, PA 15260, USA; 3 School of Health and Rehabilitation Sciences, University of Pittsburgh, Pittsburgh, PA 15260, USA

**Keywords:** accessibility, discomfort, architectural barriers, suspension, wheelchair

## Abstract

Wheelchair users are exposed to whole-body vibration (WBV) when driving on sidewalks and in urban environments; however, there is limited literature on WBV exposure to power wheelchair users when driving during daily activities. Further, surface transitions (i.e., curb-ramps) provide wheelchair accessibility from street intersections to sidewalks; but these require a threshold for water drainage. This threshold may induce high WBV (i.e., root-mean-square and vibration-daily-value accelerations) when accessibility guidelines are not met. This study analyzed the WBV effects on power wheelchairs with passive suspension when driving over surfaces with different thresholds. Additionally, this study introduced a novel power wheelchair with active suspension to reduce WBV levels on surface transitions. Three trials were performed with a commercial power wheelchair with passive suspension, a novel power wheelchair with active suspension, and the novel power wheelchair without active suspension driving on surfaces with five different thresholds. Results show no WBV difference among EPWs across all surfaces. However, the vibration-dose-value increased with higher surface thresholds when using the passive suspension while the active suspension remained constant. Overall, the power wheelchair with active suspension offered similar WBV effects as the passive suspension. While significant vibration-dose-value differences were observed between surface thresholds, all EPWs maintained WBV values below the ISO 2631-1 health caution zone.

## 1. Introduction

There are about 5.5 million wheelchair users in the United States (US) [1]. Wheelchairs provide independent mobility [2], comfort [3], and quality of life for people with disabilities to participate in communities [4]. However, the technology may be limited by surface conditions. For instance, multiple studies have demonstrated that manual wheelchair users are exposed to whole-body vibrations (WBVs) when driving on uneven and rough surfaces for long periods of time [5]. This exposure causes negative effects such as pain in the lower back, neck, and buttocks and increases the rate of muscle fatigue [6–8]. The International Standards Organization (ISO) Standard 2631-1: Mechanical Vibration and Shock. Part 1: Evaluation of Human Exposure to Whole Body Vibration 1 was established to assess the health concerns associated with WBV [9]. The standard defines a health guidance caution zone using the root-mean-square value of the weighted acceleration (RMS, unit: m/s^2^) and the vibration dose value (VDV, unit: m/s^1.75^). Based on an eight-hour exposure, a lower limit was defined at 0.5 m/s^2^ for RMS and 9.1 m/s^1.75^ for VDV. Research has shown that manual wheelchair users are exposed to vibration levels exceeding these standard recommendations [5] and tend to be exposed to vibration for about 13 h per day [6]. Further, the vibration levels may vary depending on different factors including the device suspension and ground surface properties (e.g., roughness [10]). Although ISO 2631-1 (1997) has served as a form of standardized guidance in many studies for WBV exposure in manual wheelchair users, such guidelines were originally derived from a vocational exposure level and do not consider the vibration exposure in everyday life. Further, there is limited research on the question of whether the standards adequately represent the shock and vibration exposure that electric power wheelchair (EPW) users experience daily in everyday environments. Many studies have measured vibration exposure in able-bodied people who sit for work; however, these values may not translate to people who sit on power wheelchairs for approximately 10 h per day, drive in the community, and perform vocational and recreational activities of daily living [11].

**Figure 1.**
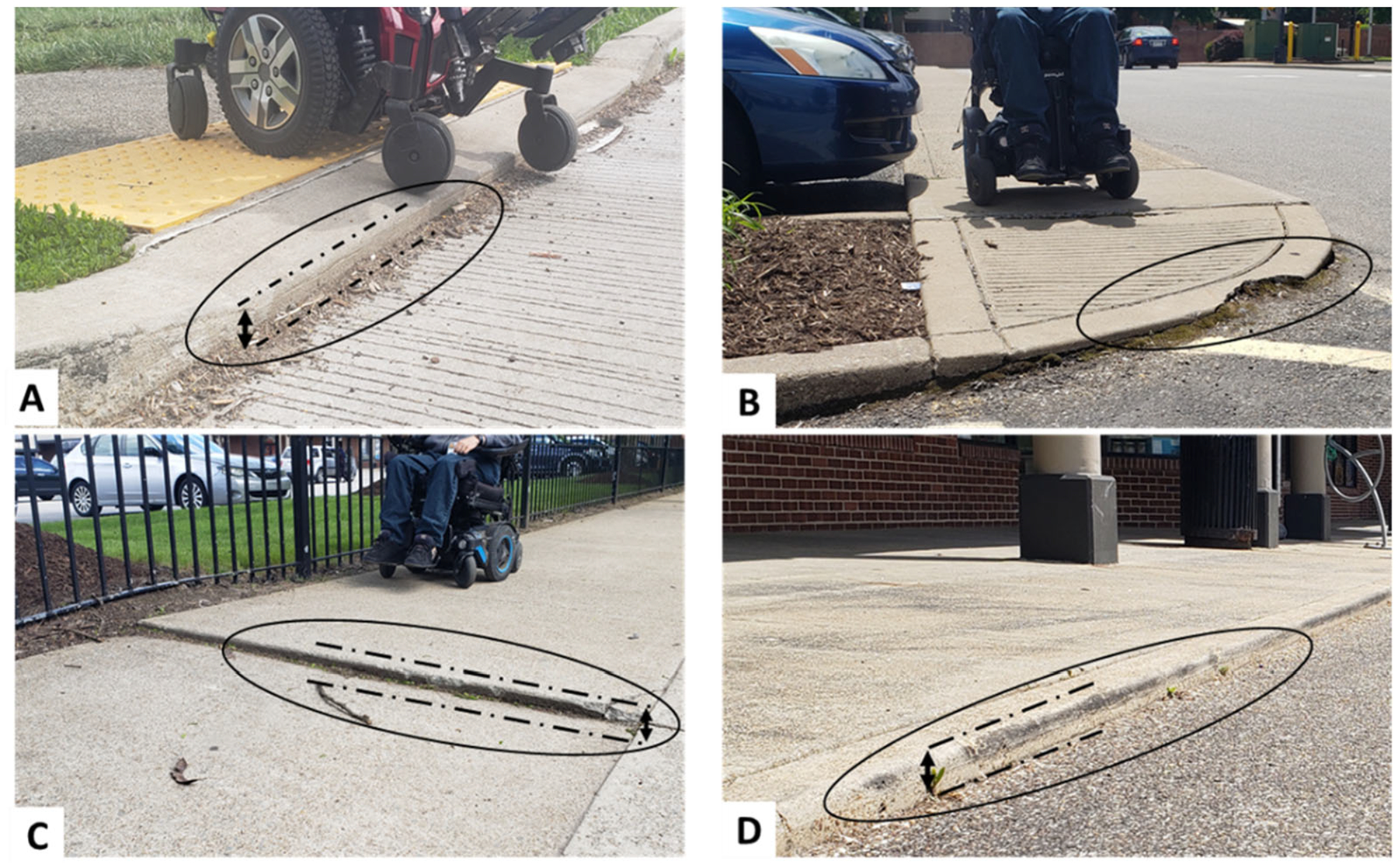
Examples of surface transitions with different thresholds at the base (illustrated with dashed lines). (**A**) curb-ramp with non-ADA complaint threshold (**B**) curb-ramp with damaged transition (**C**) sidewalk elevation (**D**) transition from sidewalk to road with high threshold.

Wolf et al. demonstrated that EPW users experience about 0.1–0.5 m/s^2^ when driving on flat surfaces composed of brick pavers and the RMS increases with surface gaps and speeds [12]. Further, Duvall et al. evaluated the RMS vibration exposure of commercial EPWs in relation to sidewalk surface roughness [10]. Their results recommended a roughness index threshold of 5.0 cm/m or an RMS vibration of 1.2 m/s^2^ for sidewalks longer than 30.5 m. These studies evaluated flat but uneven surfaces typically found on sidewalks and traversed by wheelchair users. It is important to address WBV exposure on other surfaces that EPW users commonly commute on but that may not meet the American with Disabilities Act Accessibility Guidelines (ADAAG) [13]. For instance, curb-ramps provide a surface transition between intersections and elevated sidewalks for wheelchair users. However, Bennet et al. found that only 21 out of 79 intersections in Nova Scotia, Canada had a smooth transition from curb-ramps to gutters (<1.3 cm) and only half of these meet the maximum slope requirements (<4.9°) [14] (Figure 1). The ADAAG guidelines state that obstacles should be no more than 1.3 cm in height for accessibility, regardless of flat or inclined surfaces. Driving on these surfaces may cause higher WBV magnitudes relative to smooth flat surfaces (e.g., a concrete surface). No studies were discovered evaluating the WBV exposure in EPWs on these surfaces.

Passive suspension has been widely used in manual wheelchairs and EPWs due to its low cost, simple structure (i.e., fixed spring and damper), and ability to absorb vibrations induced by the road conditions. Despite its benefits, Lariviere et al. highlighted the inconclusive results of passive suspension in manual wheelchairs. Cooper et al. found that the addition of suspension caster forks reduced the amount of vibration in manual wheelchairs when driving on small bumps of 1.3 cm by a factor of two to three [15]; the RMS values were below the health caution safety zone for a 1-h exposure for frequencies below 10 Hz. In addition, Hashizume showed similar results when driving manual wheelchairs on curbs of up to 5 cm [16]. Evaluation of passive suspension has been limited to manual wheelchairs on different surface thresholds; hence, there is a need to evaluate WBV exposure in EPWs on similar surface conditions.

An alternative to passive suspension is the use of an active suspension system that uses extra actuators together with passive suspension elements (e.g., springs and dampers) to effectively dismiss forces from the road excitation [17]. This type of suspension is used more often in commercial vehicles to increase the car’s stability and ride comfort on uneven terrains and ongoing research focuses on suspension control to reduce cost and power consumption [18]. Very few studies have proposed active suspension in EPWs; however, these were limited to simulations without a real-prototype [19,20].

The aims of this study are:

- To analyze WBV exposure in a commercial EPW, using passive suspension, when traversing different surfaces thresholds.
  - EPWs incorporate a similar suspension to manual wheelchairs in addition to their weight; hence we hypothesize that WBV measures in EPWs would be over the health safety zone of 1-h exposure on surface thresholds.
  - Additionally, WBV measures may increase with threshold height.
- To compare different types of suspension systems in EPW to minimize WBV exposure on different surface thresholds.
  - With the assumption that EPWs are exposed to vibration levels over the health guidance caution zone; we proposed a novel EPW with active suspension to ameliorate vibration exposure when facing different surface thresholds.
  - Alternatively, we hypothesized that active suspension in EPWs may reduce vibration and increase comfort on surface thresholds compared with the passive suspension presented in commercial EPWs.

## 2. Materials and Methods

### 2.1. Instrumentation

**Figure 2.**
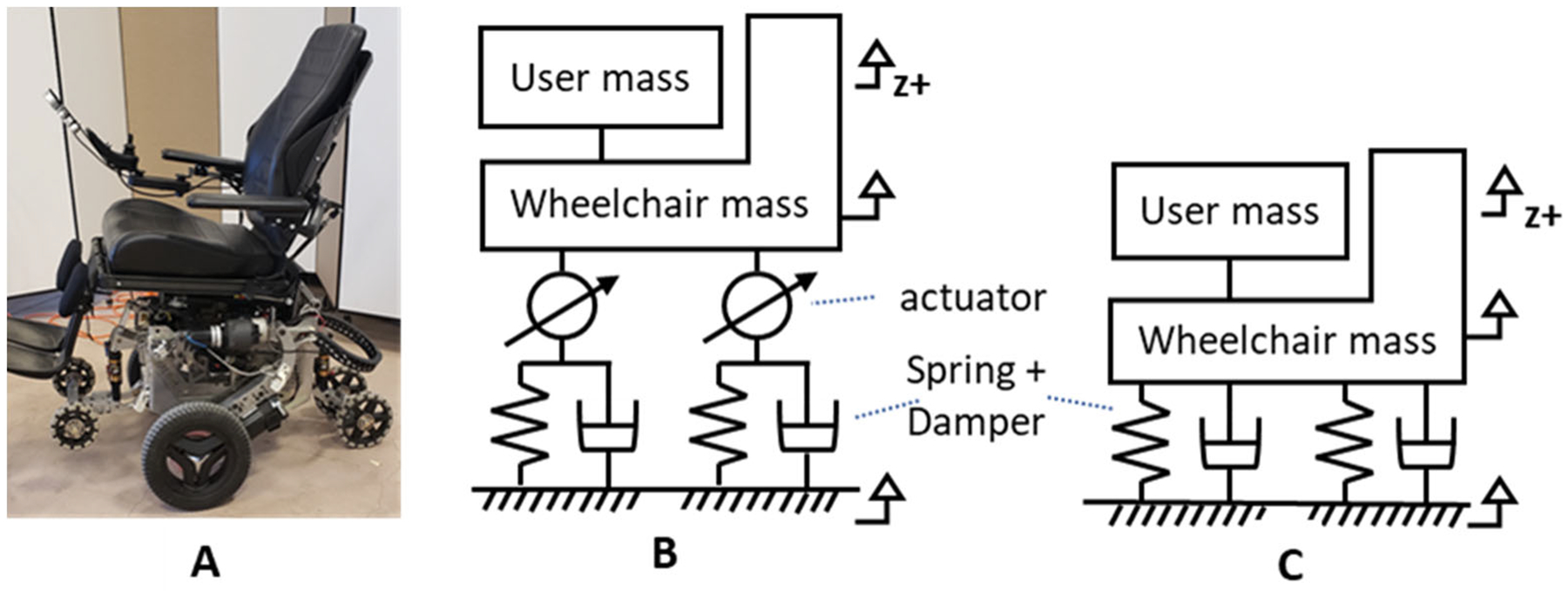
(**A**) MEBot wheelchair. (**B**) Front View of the MEBot active suspension model and (**C**) the commercial EPW passive suspension model. Wheels were considered rigid bodies.

MEBot consists of six independently height-adjustable wheels with a modular drive-wheel configuration, omni-wheels as caster wheels to eliminate swivel, and a footprint comparable to that of commercially available EPWs [21]. Each wheel was linked to an active suspension (AS) system that included an adjustable pneumatic shock absorber and an electro-hydraulic motor in series (Figure 2A,B). Shock absorbers provided a passive suspension to reduce vibration on uneven surfaces similar to EPWs; while electro-hydraulics were automatically controlled to maintain stability when surface irregularities (e.g., inclined surfaces) were detected. We hypothesize that electro-hydraulics might reduce WBV in conjunction with shock absorbers when driving on surfaces transitions that combine an inclined surface with a threshold. This study compared MEBot with active suspension (MEBot-AS), MEBot without active suspension (MEBot-noAS), and a commercial EPW with passive suspension.

MEBot-noAS refers to inhibited electro-hydraulic actuators and is only reliant on its shock absorbers. The shock absorbers were air-pressured, adjustable, and set at 100 psi per wheel. The selected commercial EPW was the Permobil F5 Corpus, a front-wheel-drive EPW with shock absorbers (Figure 2C) in each wheel to ameliorate WBV exposure [22]. EPWs with a front-wheel-drive configuration assist with obstacle climbing, stability, and traction outdoors [23]. Both EPWs used an R-Net controller to configure the same driving parameters (i.e., speed and acceleration).

**Figure 3.**
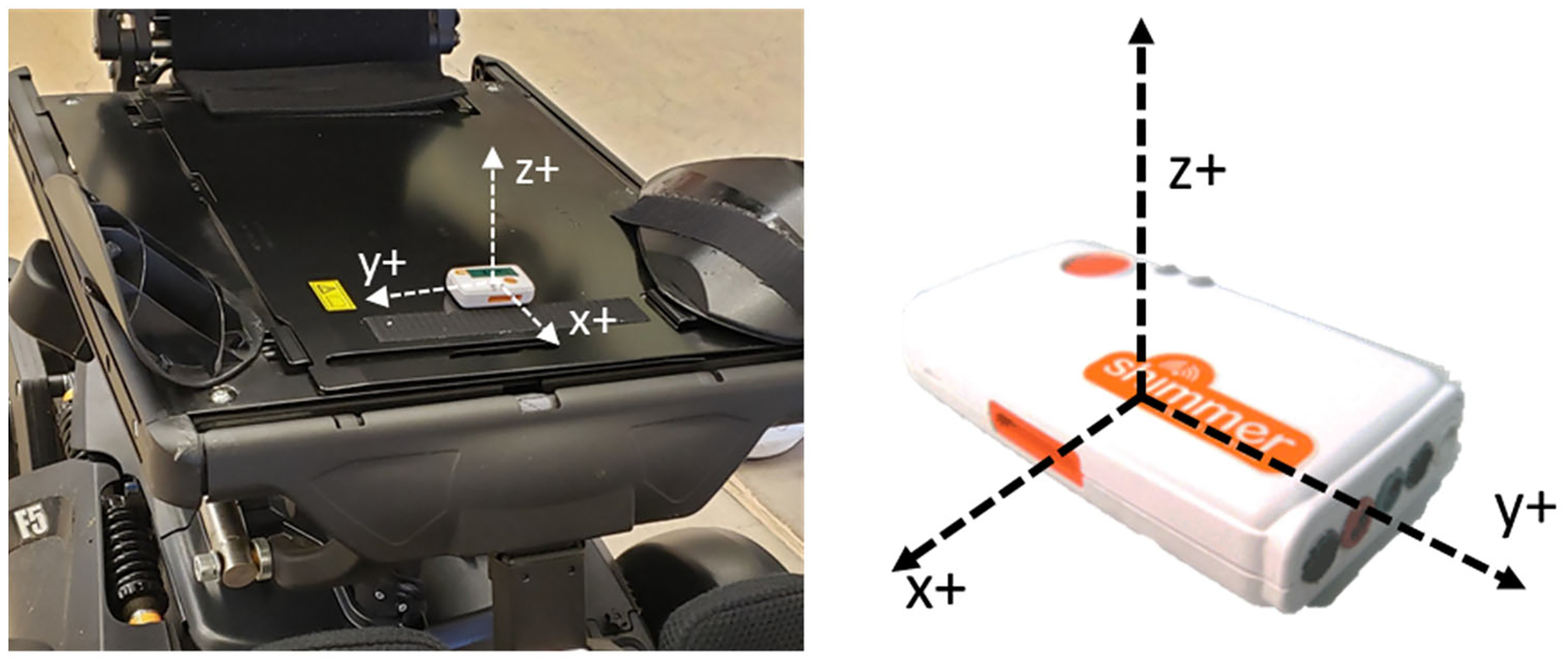
(**Left**) Shimmer 3 Triaxial accelerometer placed on the seat pan of the tested EPWs. (**Right**) Orientation of Shimmer 3 accelerometer with respect to seat pan.

The Shimmer 3 triaxial accelerometer (Shimmer, Boston, MA, USA) was mounted in the seat pan of each EPW with its z+ axis facing orthogonal to the seat as shown in Figure 3. The accelerometer incorporates a stand-alone microcontroller (STMicro LSM303AHTR) with a 14-bit resolution, high sensitivity (to detect +/−8 g), and at a sampling frequency of 100 Hz. The sampling frequency was selected in order to identify a suitable range of frequencies between 0.01 and 80 Hz according to the ISO 2631:1 standard. Similar studies acquired vibration data through accelerometers at a sampling frequency between 50 and 102 Hz [5,6,10]. The sensor was validated for use in human health monitoring, monitoring activities of daily living, and environmental and habitat monitoring [24,25].

Its accelerations were used to calculate the RMS and VDV values following the ISO 2631:1 (1997) standard. A rehabilitation seating cushion was used following the ISO 16840-2 wheelchair seating standards. The cushion material was made of high-density foam developed to support bariatric loads and to manage tissue integrity. The cushion was previously used in Garcia’s and DiGiovine’s studies, which showed a transmissibility coefficient of 1.2 and 0.5, respectively [6,26]. The cushion transmissibility was approximated to 1; therefore, the seat pan and cushion showed similar WBV exposures. Additionally, the accelerometer was placed under the seat cushion to prevent it from moving during testing and to measure vibrations directly from the rigid body.

### 2.2. Protocol

A 50th percentile Hybrid II anthropometric dummy of 100 kg was used to simulate a person seated in each EPW. Three trials were performed by driving each EPW on five selected surfaces for a total of 45 trials. Each EPW was controlled remotely by a researcher. The wheelchair speed was set to 1.2 m/s, which is the same as an average person’s speed when walking across the street [27]. A MATLAB Graphical User Interface (GUI) was developed to measure the time-series accelerations in real-time during the completion of each trial. The GUI facilitated data collection by connecting to the accelerometer, recording data, and saving it to a custom filename.

### 2.3. Surfaces

**Figure 4.**
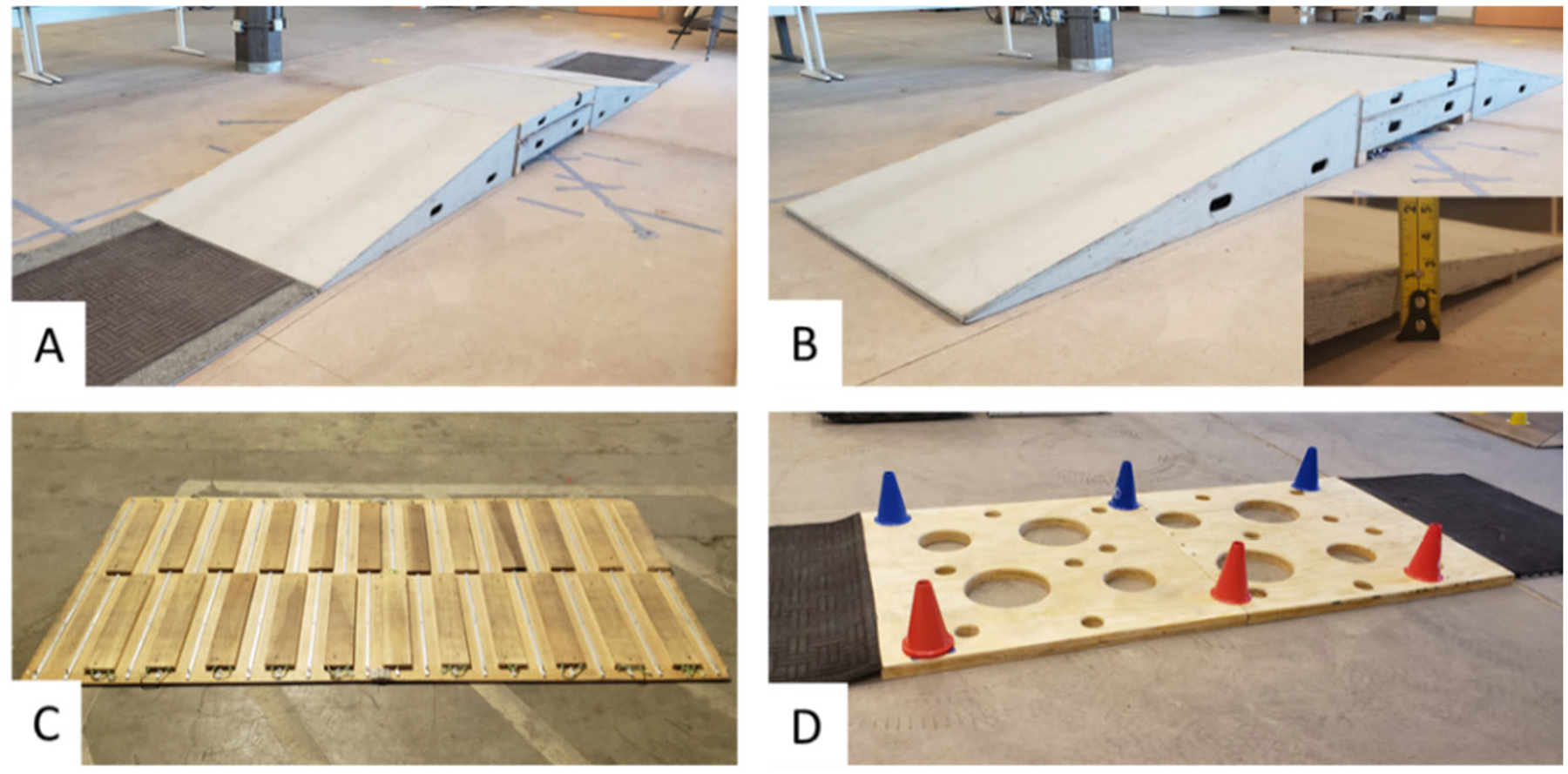
(**A**) Up-Flat-Down 10° Ramp, (**B**) Up-Flat-Down 10° Ramp with a 2.5 cm threshold in transition, (**C**) Surface roughness with adaptable slabs, and (**D)** Potholes of 5.4 cm in depth.

Five engineered driving surfaces were selected to represent surface transitions that EPW users commonly encounter daily. The tasks included: going up and down a 10° slope with and without a 2.5 cm threshold (Figure 4A,B), surfaces with a roughness of 12.5 cm/m and 18.3 cm/m (Figure 4C), and a series of potholes of up to 30.5 cm in diameter and 5.0 cm in depth (Figure 4D). The 10° slope simulated conventional incline and decline ramps considered worst-case scenarios for wheelchair dynamic stability as part of the ANSI/RESNA wheelchair standards ISO 7176-2 [28].

The slopes measured 3.1 m in length and 1.2 m in width. The 2.5 cm surface threshold simulated non-ADA thresholds obtained from Bennet’s study that reported a curb-ramps threshold of 1.9 ± 0.1 cm [14]. The ADAAG guidelines recommend a maximum 0.6 cm threshold in lip height for water drainage. Two surfaces of 12.5 cm/m and 18.3 cm/m in roughness represented uneven sidewalks and rough terrains. Both tasks were 1.2 m wide by 2.4 m long and used wooden slabs of 1.9 cm in height [10]. Last, a series of potholes were simulated based on Kirby’s wheelchair skills test v.4.1 that included 5.0 cm deep potholes across an 2.4 m long by 1.2 m wide surface [29]. The surface represented potholes caused by wear-and-tear due to weather conditions and constant use by heavy-load vehicles to which wheelchair users are exposed [30].

### 2.4. Data Analysis

Descriptive analysis (e.g., means, standard deviation) and bar graphs described the WBV parameters of each EPW in terms of RMS and VDV. These variables were calculated using the raw acceleration data obtained from the triaxial accelerometer. The raw acceleration data (*a*_*x*_*, a*_*y*_*, a*_*z*_) were first multiplied by their frequency weighting in terms of comfort (*k*_*x*_ = *k*_*y*_ = *k*_*z*_ = 1). The study focused on vibration values in the seat pan because the back rest and footplate were fixed to the seat and we considered the seat as a rigid body. The weighted accelerations were then calibrated and processed for analysis using MATLAB R2021. The first and last second time-stamp of the data were cut-off to eliminate zero values. Then, the raw accelerations in three axes were calibrated by subtracting the mean of each axis from its corresponding value. The total magnitude of each acceleration was calculated and used in the data analysis for this study. The total acceleration was viable to use because WBVs have an effect in multiple directions. Previous studies were limited in only looking at accelerations in the vertical (or gravity) direction [5]; however, this limits the analysis to only vertical accelerations and not lateral and front/back accelerations. The RMS and VDV were calculated using the total calibrated acceleration with the equations below:

(1) $$a_{\text{Total}}={\left(k_{x}^{2}a_{x}^{2}+k_{y}^{2}a_{y}^{2}+k_{z}^{2}a_{z}^{2}\right)}^{\frac{1}{2}}$$

(2) $$RMS={\left[\frac{1}{T}\int_{0}^{T}a_{\text{Total}}{\left(t\right)}^{2}dt\right]}^{\frac{1}{2}}$$

(3) $$VDV={\left[\int_{0}^{T}a_{\text{Total}}{\left(t\right)}^{4}dt\right]}^{\frac{1}{4}}$$

where *T* is the duration of the trials.

Statistical analyses were performed using the IBM SPSS software version 24.0 (SPSS, Inc., Chicago, IL, USA). Data were analyzed for normality using the Shapiro Wilk test. One-way ANOVA was performed to compare the RMS and VDV mean differences between surfaces transitions within each EPW to evaluate the effects of a surface threshold/gradient. The same statistical test was performed to compare the RMS and VDVD mean differences between EPWs for each surface transition to evaluate EPWs’ suspensions. The level of significance was set at α = 0.05 for all comparisons. If results were significant, post-hoc analysis was performed with a Bonferroni correction to adjust for multiple comparisons.

## 3. Results

**Figure 5.**
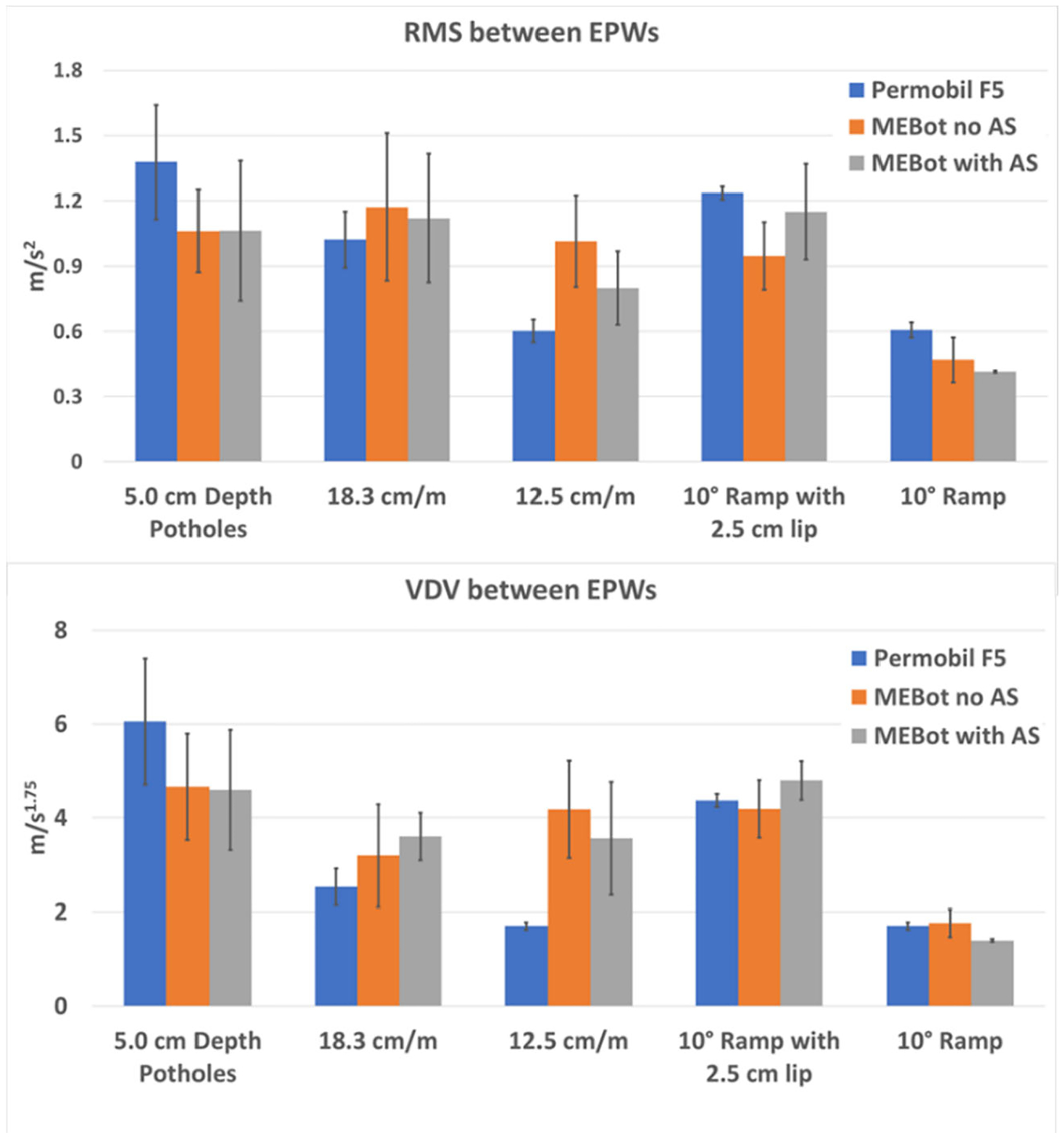
RMS total acceleration (**Top**) and VDV (**Bottom**) differences between EPWs in each surface.

Results show no significant differences in average WBV (RMS and VDV) values between the commercial EPW, MEBot with AS, and MEBot with noAS when driving on surface transitions with different thresholds (Figure 5).

**Figure 6.**
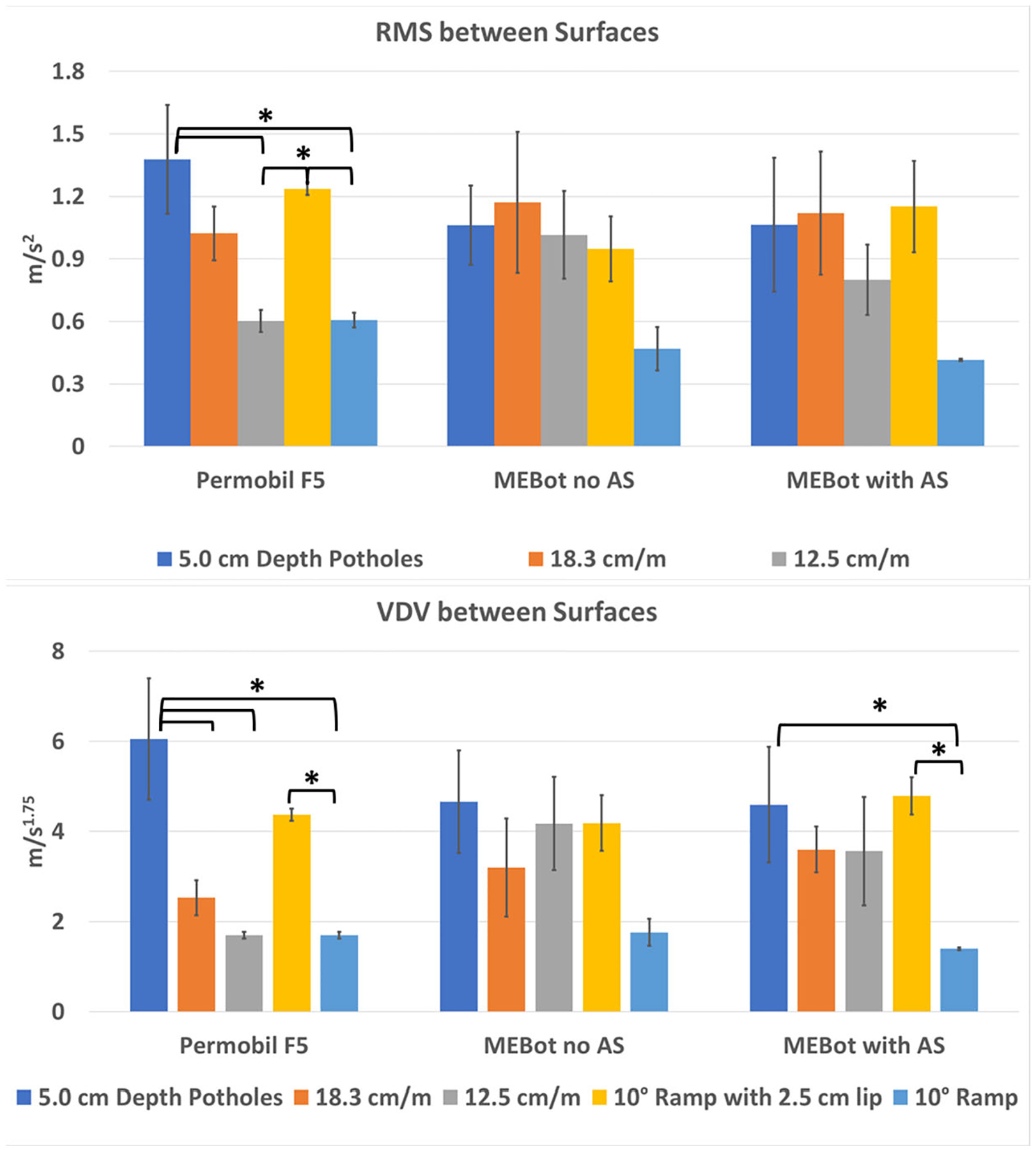
RMS total acceleration (**Top**) and VDV (**Bottom**) differences between surfaces with each EPW. Significant differences between surfaces are denoted with an asterisk (* *p*-value < 0.01 post-hoc Bonferroni correction).

In terms of WBV differences between surfaces, it was found that potholes caused significantly higher RMS values on the commercial EPW compared with the 12.5 cm/m surface roughness (*p* < 0.001) and the 10° ramp without threshold (*p* < 0.001) (Table 1). Additionally, the 10° ramp with a threshold showed higher RMS values on the commercial EPW compared with the 12.5 cm/m surface roughness (*p* = 0.002) and the 10° ramp without a threshold (*p* = 0.002). However, there were no significant RMS differences between surfaces when using MEBot with or with no AS (Figure 6).

The VDV differences between surfaces in each EPW were more noticeable with an increase in the surface threshold. The commercial EPW and MEBot with AS showed significantly lower VDV values when driving on the 10° ramp without a threshold compared with potholes of 5.0 cm in depth (*p* < 0.001). Likewise, the Permobil F5 and MEBot with AS showed significantly higher VDV exposure when driving on the 10° ramp with a 2.5 cm threshold compared with no threshold (*p* < 0.001). Additionally, the surface with potholes reported significantly higher VDV values compared with surface roughness with a 1.9 cm threshold (*p* < 0.001). No significant WBV differences were found when driving MEBot with no AS across all surfaces.

## 4. Discussion

### 4.1. WBV Differences between Surfaces

The results showed that surfaces with a threshold over 2.5 cm showed high RMS values over 1.2 m/s^2^ and a maximum RMS of 1.7 m/s^2^ when driving a commercial EPW. The ISO 2631-1 standard suggests that a RMS acceleration of 1.6 m/s^2^ or greater could be harmful over a 1-h period. This is approximately the daily duration that wheelchair users drive their assistive devices each day [31]. This finding suggests that the passive suspension of commercial EPWs and MEBot is beneficial to reduce vibration exposure on surfaces with different thresholds.

It is worth noting that the Permobil F5 is a high-end EPW with a front-wheel-drive configuration and all-terrain wheels designed to traverse environments with a threshold of up to 3.0 in. in height, according to the manufacturer. The availability of high-end EPWs as such depends on the user’s level of impairment and insurance coverage [32]. Alternative cost-effective EPWs have less weight for easier transportation but are limited to fewer seating features and less efficient drive motors. Additionally, the suspension dampening required to ameliorate WBV effects, particularly on these surfaces, is unknown. For example, Wolf et al. [12], evaluated an EPW (i.e., the Quickie P200) on flat paved surfaces that showed low RMS values within the health caution zone; however, the tested device is no longer on the market. Further evaluation of WBV effects on EPWs is encouraged to reduce EPW users’ discomfort on surfaces with thresholds.

The MEBot EPW with active suspension was introduced in this study as an alternative suspension mechanism that combines a shock absorber and an electro-hydraulic actuator in series. There were no significant RMS differences between surface thresholds whether using MEBot with or without the AS mechanism. The vibration for each surface remained below 1.2 m/s^2^ expect for potholes and the 10° ramp with a 2.5 cm threshold. These results demonstrated that MEBot can reduce the vibration with the use of shock absorbers alone; on the other hand, the use of actuators in MEBot for active suspension remains important to maintain stability on inclined and uneven surfaces to reduce tips and falls as shown by Sivakanthan et al. [33].

The findings showed that VDV results values increased with the surface threshold. For instance, the surface with potholes of 5.4 cm in depth showed significantly higher VDV values than the 10° ramp (with no threshold) and surface roughness with a 1.9 cm threshold. These results are consistent with those of the Permobil F5 and MEBot with AS. The VDV is more sensitive to the acceleration peaks; therefore, it is recommended that the amount of vibration for the case of inherent shock exposure be estimated [9]. Further, these results are consistent with a study that evaluated an EPW at a speed range of 0–0.8 m/s on a 3.6 cm threshold and showed a VDV range of 0.7–2.25 m/s^1.75^, respectively [34]. While the EPW speed was constant at 1.5 m/s, it is expected that faster speeds will increase the likelihood of higher VDV exposure. EPW users tend to avoid surfaces with high thresholds to avoid discomfort and reduce the risk of tipping or falling; however, these surfaces may be inevitable when alternative routes are not accessible nor available. Further studies should look into automatically changing the speed of the EPW on high WBV surfaces to reduce RMS and VDV values.

Further, the 10° ramp with a 2.5 cm threshold showed high VDV values compared with no threshold (Figure 6). Although, the results were below the health vibration zone of 9.0 m/s^1.75^, Bennet et al. reported a high number of curb-ramps that did not meet the ADA standards of a maximum threshold of 0.6 cm usually found in real-world conditions for water drainage [14]. Driving on these thresholds exposes EPW users to muscle pain and discomfort and can damage the assistive device over time. Damaging EPW users’ only source of assistive mobility limits their participation in the community and renders them unable to perform leisure and vocational activities. It is worth mentioning that development of city infrastructure is recommended for wheelchair user accessibility and independence. In addition, adequate settings in the EPW such as shock absorber dampening, drive wheel suspension [35], and cushioning [6] are recommended to reduce discomfort and WBV exposure when environmental barriers are present. Future studies should investigate what additional mechanisms could help reduce WBV not currently in EPWs.

### 4.2. WBV Differences between EPWs

The results show no significant RMS and VDV differences between EPWs’ suspensions across all surface thresholds and these were below the health caution zone of 1.6 m/s^2^ over 1 h of exposure at the comfort level. EPWs serve as a means of mobility for users to commute from home to work/school/shops, particularly when public or private transportation is not available or accessible [36]. The typical EPW user can drive at least 1 h/day assuming a normal speed of 1 m/s between locations [37]. During travel, EPW users may drive on sidewalks and roads with a threshold of over 2.5 cm in height. Further, EPW users are more exposed to these surface thresholds on sidewalk elevations due to tree growth on paved sidewalks and a lack of maintenance [38]. While WBV values remained below the health risk threshold, these values can increase with additional elements within the EPW such as speed, cushioning, longevity, and weight.

Figure 5 shows high WBV variance in MEBot with AS and no AS across all surfaces. A possible cause is the low dampening settings of the shock absorbers, which caused a high degree of displacement of its suspension. Likewise, a delay in the activation of the legged-wheel actuators in the AS system may have caused the EPW to replicate the surface profile, causing a bounce effect. On the other hand, the WBV variance in the EPW was only noticeable when driving on the surface with 5.0 cm potholes. Additionally, the crash dummy also plays a passive role compared with a real end-user who may intentionally correct his/her posture and, hence, reducing the WBV variance.

The active suspension of MEBot did not reduce nor increase the vibration effects when traversing surface thresholds. MEBot-AS was designed to prevent tips and falls when driving on inclined surfaces by adjusting its legged-wheel actuators in the base. Likewise, the goal of the shock absorbers was to serve as a form of passive suspension to reduce vibration [33]. The results only demonstrated that its actuators can be inhibited when driving on surfaces with thresholds to improve power consumption while prioritizing shock absorbers in these surface conditions.

### 4.3. Limitations

The study was conducted within lab settings and in controlled environments. Real-world surfaces may be affected by wear-and-tear due to weather conditions and pedestrian/vehicle traffic that we may not have included. Additionally, the wheelchair and user might be exposed to other sources of vibrations, such as large vehicles [39] and construction sites [40].

A constant speed was used, which might not be typical when facing these types of surfaces. In addition to surface thresholds, driving at a faster speed may induce higher WBV magnitudes [12]. EPW users tend to slow down when facing irregular and unfamiliar surfaces [41]. These factors should be observed in further studies on WBV exposure in EPWs and other mobility assistive devices.

The commercial EPW used had a front-wheel-drive configuration that is mostly used for active wheelchair users in the community. However, there are other drive wheel configurations (mid- and rear-wheel drive) where WBVs may have different effects on EPW users. For example, mid-wheel drive provides high stability on flat surfaces and a small turning radius, but it is at risk of getting stuck on small thresholds and ramps. Rear-wheel drive is a less common in EPWs and mostly used outdoors due to its fast speed but it is prone to tipping as its center of mass is located towards the back and its front wheels may be smaller than the suggested ADA thresholds.

Finally, EPW users were not recruited for the study to avoid WBV exposure. A crash test dummy was used to prevent discomfort to end-users and for safety when operating the EPWs on challenging surfaces. Additionally, using a crash test dummy provided control over other factors that may influence the vibration exposure, such as weight shifting, repositioning, and weight distribution, commonly encountered with end-users. On the other hand, EPW users can provide feedback in terms of health and comfort when exposed to the vibration levels on surface transitions. Their feedback is important to be able to offer the most adequate mobility assistive device to reduce WBV exposure. Further studies may include subject testing to evaluate the feasibility of EPW suspensions.

## 5. Conclusions

This study aimed to explore the WBV effects in EPWs when encountering challenging surfaces. While many studies have evaluated vibration in manual wheelchairs, there are few studies that evaluate the vibration effects in EPWs, particularly on surfaces with thresholds that end-users are exposed to daily. To the best of our knowledge, this is the first study to evaluate EPW suspensions on surfaces with different thresholds (heights) such as uneven sidewalks and curb-ramps that are not ADA-compliant. Likewise, this is the first study to compare two types of EPW suspension systems (passive and active suspension) to reduce WBV measures on the selected surfaces. The study introduced a novel EPW with active suspension to increase stability and the user’s comfort. The results show similar WBV values that lie within the health guidance safety zone; therefore, no difference was found between passive and active EPW suspension. The study also demonstrated a proportional increase in RMS and VDV values with the surface threshold when using the EPW passive suspension compared with the EPW active suspension, which demonstrated constant vibration values in all surface thresholds. The results of this study will increase the amount on literature of WBV exposure in EPWs. This study was also conducted with a crash dummy and in controlled environments for safety reasons. Further evaluation of EPW suspension systems should include end-users to obtain their perception of comfort and health with respect to the WBV exposure in every day environments.

## Figures and Tables

**Table 1.** Root-mean-square (RMS) and vibration dose value (VDV) of tested devices per surface.

Surfaces	Devices	RMS (m/s^2^)	VDV (m/s^1.75^)
Potholes 5.0 cm in depth	Permobil F5	1.4 ± 0.3	6.1 ± 1.3
	MEBot no AS	1.1 ± 0.2	4.7 ± 1.1
	MEBot with AS	1.1 ± 0.3	4.6 ± 1.3
18.3 cm/m Surface Roughness	Permobil F5	1.0 ± 0.1	2.5 ± 0.4
	MEBot no AS	1.2 ± 0.3	3.2 ± 1.0
	MEBot with AS	1.1 ± 0.3	3.6 ± 0.5
12.5 cm/m Surface Roughness	Permobil F5	0.6 ± 0.1	1.7 ± 0.1
	MEBot no AS	1.0 ± 0.2	4.2 ± 1.0
	MEBot with AS	0.8 ± 0.2	3.6 ± 1.2
10° Ramp with 2.5 cm threshold	Permobil F5	1.2 ± 0.0	4.4 ± 0.1
	MEBot no AS	0.9 ± 0.2	4.2 ± 0.6
	MEBot with AS	1.2 ± 0.2	4.8 ± 0.1
10° Ramp No Threshold	Permobil F5	0.6 ± 0.0	1.7 ± 0.1
	MEBot no AS	0.5 ± 0.1	1.8 ± 0.3
	MEBot with AS	0.4 ± 0.0	1.4 ± 0.0

## Data Availability

The data presented in this study are available on request from the corresponding author. The data are not publicly available due to their being restored by the US Department of Veterans Affairs and subject to the approval of the relevant authority.
